# Association Between Nutritional Biomarkers and Low Muscle Mass, Obesity, and Low Muscle Mass with Obesity Across Physical Activity Levels Among U.S. Adults: Finding from the National Health and Nutrition Examination Survey 2015–2018

**DOI:** 10.3390/ijerph23060815

**Published:** 2026-06-19

**Authors:** Uraiporn Booranasuksakul, Mario Siervo, Alongkote Singhato, Narisa Rueangsri, Tepparit Samrit, Wichukorn Suriyawongpaisal, Piyapong Prasertsri

**Affiliations:** 1 Faculty of Allied Health Sciences, Burapha University, Chon Buri 20131, Thailand; uraiporn@buu.ac.th (U.B.); alongkote@go.buu.ac.th (A.S.); narisar@go.buu.ac.th (N.R.); tepparit.sa@go.buu.ac.th (T.S.); 2 Curtin School of Population Health, Faculty of Health Sciences, Curtin University, Bentley, WA 6102, Australia; mario.siervo@curtin.edu.au; 3 ASEAN Institute for Health Development, Mahidol University, Nakhon Pathom 73170, Thailand; wichukorn.sur@mahidol.ac.th

**Keywords:** low muscle mass, obesity, low muscle mass with obesity, nutritional biomarker, physical activity

## Abstract

**Highlights:**

**Public health relevance—How does this work relate to a public health issue?**

- Low muscle mass, obesity, and low muscle mass with obesity were strongly associated with adverse nutritional, metabolic, and inflammatory biomarkers among U.S. adults.
- Physical inactivity was associated with a higher prevalence of unfavorable body composition phenotypes and cardiometabolic risk biomarkers.

**Public health significance—Why is this work of significance to public health?**

- This study highlights the combined impact of low muscle mass and obesity on insulin resistance and systemic inflammation in a nationally representative population.
- The findings suggest that sufficient physical activity was associated with lower adverse links between nutritional biomarkers and body composition abnormalities.

**Public health implications—What are the key implications or messages for practitioners, policy makers and/or researchers in public health?**

- Public health strategies promoting regular physical activity and nutritional health may help prevent low muscle mass, obesity, and related metabolic disorders.
- Screening nutritional and inflammatory biomarkers alongside body composition assessment may improve early identification of individuals at risk for sarcopenic obesity and cardiometabolic complications.

**Abstract:**

Background: Nutritional biomarkers are linked to body composition changes, but limited evidence has studied how nutritional biomarkers relate to low muscle mass, excess adiposity, and both coexisting conditions across different physical activity levels. This study aims to investigate associations between low muscle mass, obesity, and low muscle mass with obesity and nutritional biomarkers across physical activity levels among U.S. adults across physical activity levels. Methods: This cross-sectional study analyzed data from adults aged 20–59 years from the 2015–2018 cycles of the National Health and Nutrition Examination Survey (NHANES) 2015–2018. Low muscle mass was defined by low appendicular lean mass relative to body weight (LALM/W). Obesity was classified using body mass index (BMI^1^), waist circumference (WC^2^), and body fat percentage (FM%^3^), and low muscle mass with obesity was defined using three coexisting phenotypes (LALM/W-O1, LALM/W-O2, LALM/W-O3). Nutritional biomarkers included serum albumin, vitamin D, triglyceride, cholesterol, LDL cholesterol, iron, insulin resistance (HOMA IR), and high-sensitivity C-reactive protein (hs-CRP). Physical activity was categorized as inactive, insufficiently active, or sufficiently active based on MET minutes per week. Multivariable regression models accounted for the complex survey design and relevant covariates. Results: After adjustment, LALM/W was significantly associated with low serum albumin, low vitamin D, high triglyceride, high HOMA-IR, and high CRP. Obesity was significantly associated with low serum albumin, low vitamin D, high triglyceride, high LDL cholesterol, high HOMA-IR, and high CRP. LALM/W-O in all phenotypes were significantly associated with low serum albumin, low vitamin D, high triglyceride, high LDL cholesterol, high HOMA-IR, and high CRP. LALM/W-O phenotypes demonstrated the strongest associations, particularly with high HOMA-IR and hs-CRP. Although the associations varied by physical activity level, sufficiently active group was associated with lower odds of adverse nutritional biomarkers compared with insufficient activity. Conclusions: Nutritional biomarkers are associated with LALM/W and obesity. Sufficient physical activity was associated with fewer adverse outcomes. This suggests that adequate physical activity may be associated with better nutritional status and body composition.

## 1. Introduction

Sarcopenia, defined as the progressive loss of muscle mass, muscle strength, and muscle function, is a major public health concern due to its association with a higher incidence of falls, new or prolonged hospitalizations, fractures, loss of mobility and physical function, and mortality [1]. Low muscle mass is commonly observed in younger adults [2]. The prevalence of low muscle mass was significantly elevated among the age group of 18–39 years old (from 11.3% to 14.1% [3]. Concurrently, obesity continues to be very common, contributing to metabolic disorders and chronic inflammation [4,5]. Sarcopenia and obesity share common underlying mechanisms, including chronic low-grade inflammation, insulin resistance, oxidative stress, and hormonal alterations [6]. These overlapping pathways emphasize the importance of examining both conditions together rather than in isolation.

Nutrition status is an important factor of the pathogenesis process that includes muscle metabolism, adiposity, and systemic inflammation [7,8]. Abnormal levels of nutritional and metabolic biomarkers, including serum albumin, vitamin D, lipid profiles, insulin resistance, and inflammatory markers, have been associated with poor muscular function, obesity, and metabolic syndrome [8]. The presence of indicators of insulin resistance (e.g., homeostatic model assessment for insulin resistance) has been significantly correlated with both sarcopenia and obesity [9].

Physical activity is another contributing factor to muscle health and obesity that may modify the link between nutritional biomarkers and body composition profiles. Physical inactivity has been associated with muscle loss and fat accumulation, which have been linked to sarcopenia and obesity. In turn, sarcopenia and obesity have been associated with impaired exercise capacity and physical function, suggesting a bidirectional relationship between these conditions [10]. Physical activity is linked with increased muscle mass, reduced inflammation, and improved metabolic health, while sedentary behavior leads to muscle loss and fat gain [11]. However, limited research has examined how associations between nutritional biomarkers and body composition phenotypes vary across physical activity levels, particularly in nationally representative populations.

Therefore, this study aims to investigate the association between low muscle mass, obesity, and low muscle mass with obesity among U.S. adults and nutritional biomarkers. Furthermore, it explores whether these associations differ across levels of physical activity, providing a more comprehensive understanding of the interaction among nutrition, body composition, and physical activity.

## 2. Materials and Methods

### 2.1. Study Design and Population

This cross-sectional study applied data from the NHANES 2015–2018 cycles, a nationally representative survey of the U.S. population conducted by the Centers for Disease Control and Prevention. NHANES uses a complex, multistage probability sampling design to collect demographic, clinical, laboratory, and examination data. Participants aged 20–59 years with complete data on body composition, anthropometry, nutritional biomarkers, and physical activity were included in the analysis. Participants with fasting subsample weights from NHANES were included in the analysis. Individuals with missing key variables were excluded.

### 2.2. Ethical Considerations

Ethical approval was not required for this study because it involved a secondary analysis of publicly available de-identified data. The original NHANES protocols were approved by the National Center for Health Statistics (NCHS) Research Ethics Review Board, and written informed consent was obtained from all participants prior to data collection.

### 2.3. Definition of Body Composition Phenotypes

Low muscle mass was defined as the presence of low muscle mass only, without concomitant low muscle strength or impaired physical performance [2,12]. Muscle mass was assessed using appendicular lean mass (ALM) obtained from dual-energy X-ray absorptiometry (DXA), adjusted for body weight (ALM/W). LALM/W was defined as ALM/W <28.27% for men and <23.47% for women, while values above these cutoffs were classified as normal. This approach aligns with evidence from SO frameworks, such as the ESPEN–EASO consensus, which emphasize muscle mass relative to body weight as a relevant construct in adult population studies [13].

Obesity was defined using multiple criteria, including body mass index (BMI), waist circumference (WC), and body fat percentage (FM%). Obesity was classified using BMI, categorized as <18.5, 18.5–24.9, 25.0–29.9, and ≥30 kg/m^2^, with BMI ≥30 kg/m^2^ indicating obesity and 18.5–24.9 classified as normal. Abdominal obesity was defined using WC, with ≥102 cm for men and ≥88 cm for women indicating high WC, and values below these cutoffs classified as normal. Obesity based on FM% was defined as >25% for men and >32% for women, while values below these cutoffs were considered normal.

Low muscle mass with obesity (LALM/W-O) was defined as the coexistence of low muscle mass and obesity. Three definitions of LALM/W-O were applied including LALM/W-O1, LALM/W-O2, and LALM/W-O3.

LALM/W-O1: Defined as the coexistence of low ALM/W and high BMI (≥30 kg/m^2^), while those with high ALM/W and normal BMI (18.5–24.9 kg/m^2^) were classified as normal.

LALM/W-O2: Defined as the coexistence of low ALM/W and high WC, while those with high ALM/W and low WC were classified as normal.

LALM/W-O3: Defined as the coexistence of low ALM/W and high FM%, while participants with high ALM/W and low FM% were considered normal.

### 2.4. Nutritional Biomarkers

Nutritional biomarkers were obtained from laboratory data including serum albumin, serum vitamin D, lipid profile (total cholesterol, low-density lipoprotein (LDL), and triglycerides), serum iron, insulin resistance, and high-sensitivity C-reactive protein (hs-CRP). Insulin resistance was assessed using the homeostatic model assessment for insulin resistance (homeostatic model assessment for insulin resistance: HOMA-IR), calculated as fasting insulin (µU/mL) multiplied by fasting glucose (mmol/L) divided by 22.5 [14]. These biomarkers were selected to reflect nutritional status, metabolic health, and systemic inflammation.

Nutritional biomarkers were classified using cutoffs consistent with NHANES protocols and U.S. clinical guidelines: serum albumin < 3.5 g/dL indicating hypoalbuminemia [15]; vitamin D (25-hydroxyvitamin D) < 20 ng/mL indicating deficiency [16]; total cholesterol ≥ 200 mg/dL [17], triglycerides ≥ 150 mg/dL [18], and LDL ≥ 100 mg/dL [19] indicating adverse lipid profiles; serum iron <60 µg/dL indicating iron deficiency [20]; insulin resistance defined as HOMA-IR ≥ 2.5 [21]; and hs-CRP > 3 mg/L indicating high inflammatory risk according to cardiovascular risk stratification criteria [22].

### 2.5. Physical Activity Levels

Physical activity data were collected through interviewer-administered questionnaires in NHANES. Participants reported the frequency and duration of moderate and vigorous recreational activities. Total physical activity was calculated as metabolic equivalent (MET)-minutes per week based on self-reported frequency (days/week) and duration (minutes/day) of moderate and vigorous intensity from both work-related and recreational (leisure-time) domains which were collected in the NHANES. Moderate and vigorous activities were assigned MET values of 4.0 and 8.0, respectively [23], and total MET-minutes per week were computed by summing the products of frequency, duration, and corresponding MET values. MET-minutes per week was categorized into three levels: inactive (0 MET-min/week), insufficiently active (1–599 MET-min/week), and active (≥600 MET-min/week), based on recommendations from the World Health Organization. Extreme data of total physical activity were truncated at 11,520 MET-min/week to minimize potential overestimation. This threshold was derived from established physical activity data processing guidelines, which recommend a maximum of 960 min per week per activity type [24].

### 2.6. Statistical Analysis

The complex survey design of NHANES was accounted for in all analyses by incorporating sampling weights, strata, and primary sampling units, and fasting subsample weights were applied for analyses involving fasting laboratory biomarkers in accordance with NHANES analytical guidelines. Statistical analyses were performed using IBM SPSS Statistics for Windows, Version 29.0 (IBM Corp., Armonk, NY, USA). For analyses combining the 2015–2016 and 2017–2018 cycles, 4-year sample weights were constructed by dividing the 2-year sample weights by two. Nominal and ordinal data were analyzed using Complex Samples Frequencies, whereas continuous variables were analyzed using Complex Samples Descriptive statistics. Variables are presented as weighted estimates (Est) with standard errors (SE) derived from the complex sampling design. Continuous variables are reported as survey-weighted means, while categorical variables are presented as survey-weighted percentages (%).

Multivariable Complex Sample Logistic Regression Models were used to observe the associations between body composition phenotypes (LALM/W, obesity, and LALM/W-O) and nutritional biomarkers. Formal interaction analyses were conducted by including interaction terms between body composition phenotype and physical activity level. Potential confounders were selected based on the prior literature which included age, sex, race/ethnicity, educational level, smoking status, alcohol consumption, and multimorbidity (diabetes, thyroid problem, cancer or malignancy, liver condition, hypertension, coronary heart disease, heart attack, congestive heart failure, stroke, asthma, COPD, arthritis, and kidney problem).

Analyses were stratified by physical activity levels to examine potential effect modification. Adjusted models controlled for relevant covariates. Results were reported as odds ratios (ORs) with 95% confidence intervals (CIs), and statistical significance was defined as a two-sided *p*-value < 0.05.

## 3. Results

**Figure 1:**
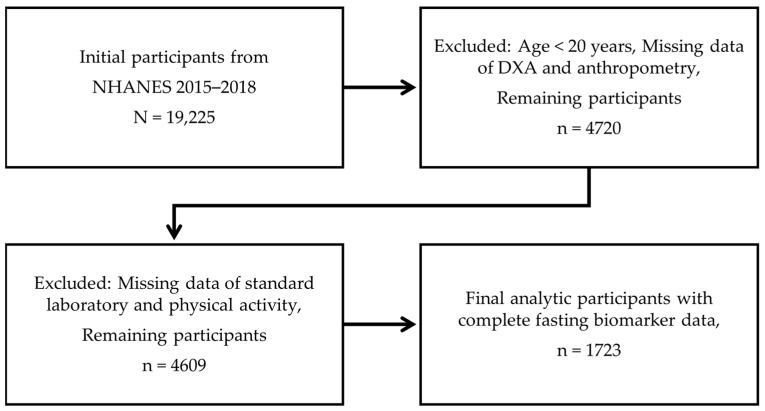
Flowchart of participant selection for the NHANES 2015–2018 study population.

The participant selection process is shown in Figure 1. From 19,225 NHANES 2015–2018 participants, 4720 participants met the initial eligibility criteria after exclusion for age and missing DXA or anthropometric data. After excluding participants with incomplete laboratory or physical activity data, 4609 participants were included in the analytic sample, of whom 1723 had complete fasting biomarker data.

The weighted sociodemographic, lifestyle, health-related, and dietary characteristics of 1723 U.S. adults from NHANES 2015–2018 according to physical activity levels (Table 1).

Overall, participants were similarly distributed between the two age groups, with 49.7% aged 20–39 years and 50.3% aged 40–59 years. The sufficiently active group had a higher proportion of younger adults aged 20–39 years (52.3%), whereas inactive group was more likely to be aged 40–59 years (55.5%), suggesting lower physical activity participation with increasing age. Women represented a greater proportion of the total sample (55.2%). Regarding race/ethnicity, non-Hispanic White participants show the largest proportion of the study population (58.1%). Socioeconomic differences were also observed. Participants with higher annual household income were more represented in the sufficiently active group (86.2%) than in the inactive group (79.7%). Similarly, individuals with lower education were more common in the inactive group (20.4%), while higher education was more prevalent among sufficiently active adults (39.7%). Smoking prevalence was highest among inactive adults (29.3%) and alcohol consumption was also higher in the inactive group (15.1%). Increased multimorbidity was most common among inactive adults (24.8%) and lowest among sufficiently active adults (19.2%). Mean energy intake was highest among sufficiently active adults (2103.6 kcal/day) and a similar trend was observed for carbohydrate intake.

Table 2 presents the weighted distribution of body composition phenotypes and nutritional biomarkers according to physical activity levels. Overall, 28.7% of participants had LALM/W, with a higher prevalence among inactive (37.5%) and insufficiently active adults (35.5%) compared with sufficiently active adults (24.5%).

Based on BMI, 37.1% of participants were classified as obesity (BMI ≥ 30 kg/m^2^). Obesity prevalence was higher in the inactive (39.1%) and insufficiently active groups (39.6%) than in the sufficiently active group (36.0%). Using WC criteria, 51.3% of participants had central obesity, with the highest prevalence in inactive adults (57.1%). High FM% was more prevalent among inactive (84.7%) and insufficiently active adults (82.7%) than in the sufficiently active group (69.3%).

Individuals with LALM/W-O1 (low ALM/W with high BMI) were observed at 19.6% overall and was highest among inactive adults (22.9%). LALM/W-O2 (low ALM/W with high WC) was 24.5% overall, with the highest prevalence in the inactive group (31.3%). LALM/W-O3 (low ALM/W with high FM%) affected 28.7% overall and was more common in inactive adults (37.5%).

Regarding nutritional biomarkers, low serum albumin was uncommon overall (1.5%). Vitamin D deficiency affected 25.3% overall and was most prevalent among insufficiently active adults (39.1%). Higher triglycerides were present at 26.3% overall, with the highest prevalence in the insufficiently active group (35.8%). High total cholesterol affected 38.8% overall and was most common among inactive adults (43.4%). Higher LDL cholesterol was present at 62.9% overall, with the highest prevalence in the inactive group (68.9%). Low serum iron was observed in 20.3% overall and was more common among inactive adults (25.1%). Insulin resistance (HOMA-IR ≥2.5) affected 43.0% overall and was more prevalent among inactive adults (48.7%). High hs-CRP was observed in 30.8% overall and was highest among inactive adults (37.6%).

Table 3 presents crude (Model 1) and fully adjusted (Model 2) odds ratios (ORs) with 95% confidence intervals (CIs) for the association between LALM/W and adverse nutritional, metabolic, and inflammatory biomarkers according to physical activity level (inactive, insufficiently active, and sufficiently active). Model 2 was adjusted for covariates. Formal interaction analyses were conducted to evaluate whether the associations between body composition phenotypes and nutritional biomarkers differed according to physical activity level (Table S1). Significant interactions with physical activity level were observed for BMI-defined obesity and LALM/W-O1 in relation to albumin, triglycerides, total cholesterol, LDL cholesterol, iron, and hs-CRP (all *p*-interaction < 0.001). In addition, significant interactions were observed between WC-defined obesity and albumin (*p*-interaction < 0.001), FM%-defined obesity and LDL cholesterol (*p*-interaction < 0.001), between LALM/W-O2 and albumin, iron, and HOMA-IR (*p*-interaction < 0.05), and between LALM/W-O3 and LDL cholesterol (*p*-interaction = 0.02). These findings indicate that the associations between these body composition phenotypes and nutritional biomarkers varied according to physical activity level. No significant interactions were observed for vitamin D, and most other phenotype–biomarker combinations showed no evidence of effect modification by physical activity level.

In the total study population (n = 1723), LALM/W was significantly associated with greater odds of low albumin, low vitamin D, high triglycerides, high HOMA-IR, and high hs-CRP, with adjusted ORs ranging from 1.6 to 3.5. The associations for total cholesterol and LDL cholesterol were associated with lower after adjustment and were no longer statistically significant. However, the findings for low albumin should be interpreted cautiously due to the very low prevalence of hypoalbuminemia (1.5%).

Among inactive adults, LALM/W was significantly associated with low albumin in the crude model (OR = 4.4, 95% CI: 1.3–15.4), although this association was lower after full adjustment. LALM/W was associated high total cholesterol within the crude model (OR = 1.7, 95% CI: 1.6–2.5) but no longer remained significant in the adjusted model. Participants with LALM/W had higher odds of higher HOMA-IR in the unadjusted model (OR = 1.9, 95% CI: 1.2–3.1), but this association was not retained after adjustment. LALM/W remained significantly associated with high hs-CRP even after full adjustment (OR = 2.2, 95% CI: 1.1–4.4).

Among insufficiently active adults, most associations were not significant, likely due to the smaller sample size. However, LALM/W showed a strong and independent association with higher HOMA-IR after adjustment (OR = 10.1, 95% CI: 3.8–27.2). Other biomarkers, including albumin, vitamin D, lipids, iron, ferritin, and hs-CRP, were not significantly associated after multivariable adjustment.

Among sufficiently active adults, LALM/W associated with several biomarkers remained significant after adjustment. LALM/W was associated with low albumin (OR = 4.6, 95% CI: 1.9–11.2), low vitamin D (OR = 2.1, 95% CI: 1.3–3.3), high triglycerides (OR = 2.4, 95% CI: 1.5–3.9), high HOMA-IR (OR = 4.6, 95% CI: 2.7–7.7), and high hs-CRP (OR = 4.1, 95% CI: 2.7–6.3).

Table 4 shows the associations of obesity phenotypes with nutritional, metabolic, and inflammatory biomarkers. In the total study population (n = 1723), all obesity phenotypes were significantly associated with greater odds of high triglycerides, high LDL cholesterol, high HOMA-IR, and high hs-CRP, with adjusted ORs ranging from 1.7 to 16.9. Obesity, defined by high BMI and high WC, were significantly associated with low albumin and low vitamin D after adjustment, but not with obesity defined by high FM%. Odds ratios for low albumin could not be estimated in FM% phenotypes, possibly because of the very low prevalence of hypoalbuminemia (1.5%), resulting in insufficient cases for stable estimation.

Across all physical activity groups, participants with BMI-defined obesity had substantially higher odds of insulin resistance (high HOMA-IR). In the fully adjusted model, obesity was associated with high HOMA-IR in inactive group (OR = 18.2, 95% CI: 8.7–38.1), insufficiently active group (OR = 43.2, 95% CI: 7.8–237.4), and sufficiently active group (OR = 15.1, 95% CI: 8.1–28.3) compared with normal BMI (18.5–24.9 kg/m^2^). High BMI was also associated with increased hs-CRP in inactive group (OR = 6.8, 95% CI: 3.2–14.4) and sufficiently active group (OR = 8.4, 95% CI: 4.7–14.8). Similarly, Vitamin D deficiency was more common among participants with high BMI and insufficiently active and sufficiently active participants. Participants with high BMI had higher odds of hypertriglyceridemia in inactive and sufficiently active groups.

Participants with high WC had significantly higher odds of high HOMA-IR across all activity groups. High WC was also associated with increased odds of high hs-CRP and high triglycerides, particularly among inactive and sufficiently active adults.

Participants with high FM% had markedly higher odds of high HOMA-IR and high hs-CRP across all physical activity levels. Associations with high triglycerides and LDL cholesterol were also observed, especially among sufficiently active adults.

Table 5 shows the associations between three definitions of LALM/W-O1-3 and adverse nutritional, metabolic, and inflammatory biomarkers according to physical activity level. In the total study population (n = 1723), all LALM/W-O phenotypes were significantly associated with greater odds of low vitamin D, high triglycerides, high LDL cholesterol, high HOMA-IR, and high hs-CRP, with adjusted ORs ranging from 1.8 to 42.1. LALM/W-O phenotypes, defined by high BMI and high WC, were significantly associated with low albumin after adjustment but could not be estimated in FM% phenotypes.

Among inactive adults, LALM/W-O1 was strongly associated with high HOMA-IR and high hs-CRP. In the fully adjusted model, participants with LALM/W-O1 had higher odds of high HOMA-IR (OR = 16.3, 95% CI: 6.1–43.3) and high hs-CRP (OR = 9.4, 95% CI: 3.6–24.7). High triglyceride also remained significantly associated with this group. Among insufficiently active adults, LALM/W-O1 showed the strongest association with high HOMA-IR. Low vitamin D and high triglycerides were also more common in this group. However, the confidence intervals were wide, likely because the subgroup sample size was small. Among sufficiently active adults, LALM/W-O1 remained significantly associated with multiple adverse biomarkers. Participants with LALM/W-O1 had 26.0-fold higher odds of elevated HOMA-IR and 15.2-fold higher odds of high hs-CRP. In addition, significant associations with high triglyceride and vitamin D deficiency were observed.

Among inactive adults, LALM/W-O2 was independently associated with high HOMA-IR (OR = 5.2) and high hs-CRP (OR = 6.0). Among insufficiently active adults, LALM/W-O2 showed an extremely strong association with insulin resistance (OR = 84.4). Although some additional biomarkers showed higher odds, statistical precision was limited due to a smaller sample size. Among sufficiently active adults, LALM/W-O2 remained strongly associated with high HOMA-IR (OR = 20.1) and high hs-CRP (OR = 9.2). Significant associations with low vitamin D, high triglycerides and high LDL cholesterol were also observed.

Among inactive adults, LALM/W-O3 was significantly associated with high HOMA-IR (OR = 12.9) and high hs-CRP (OR = 10.0). Among insufficiently active adults, LALM/W-O3 showed one of the strongest inflammatory associations in the table. Participants had 150.0-fold higher odds of elevated HOMA-IR and 34.8-fold higher odds of high hs-CRP. Although higher odds were observed, the smaller sample size resulted in wider confidence intervals and lower precision. Among sufficiently active adults, LALM/W-O3 remained independently associated with elevated HOMA-IR (OR = 23.1), high hs-CRP (OR = 11.9), low vitamin D (OR = 2.7), high triglyceride (OR = 5.8), and high LDL cholesterol (OR = 2.4).

Overall, the associations between all adverse body composition phenotypes LALM/W, obesity, and LALM/W-O) and high HOMA-IR and hs-CRP showed the strongest and most consistent associations across all physical activity levels.

## 4. Discussion

Overall, among the representative U.S. adults included in this NHANES-based study, adverse body compositions characterized by decreased lean mass and increased adiposity were associated with undesirable nutritional, metabolic, and inflammatory biomarkers. Individuals with low muscle mass showed greater odds of hypoalbuminemia, vitamin D deficiency, hypertriglyceridemia, insulin resistance, and high hs-CRP, while obesity phenotypes were additionally associated with high LDL cholesterol. These findings demonstrate that low muscle mass and obesity share common underlying metabolic and inflammatory mechanisms and link to inadequate nutritional status, metabolic dysregulation, and chronic low-grade inflammation [18,25,26,27,28,29,30]. These findings were further reinforced by the LALM/W-O phenotypes in this study, which showed that all LALM/W-O phenotypes were significantly associated with greater odds of hypoalbuminemia, vitamin D deficiency, dyslipidemia, insulin resistance, and systemic inflammation, particularly regarding insulin resistance and inflammation [30]. The associations between LALM/W-O, obesity, and LALM/W-O with hypoalbuminemia, vitamin D deficiency, and dyslipidemia further indicate that nutritional status and lipid metabolism remain essential contributors [18,19,25,26,27,28].

Regular physical activity has been associated with improvements in insulin sensitivity, lipid metabolism, body composition, and systemic inflammation, which may influence the metabolic consequences of adverse body composition phenotypes. In the previous literature, exercise has been found to potentially positively impact body composition, physical functional capacity, and metabolic profiles in individuals with SO, while also mitigating the inflammation and metabolic dysfunction due to obesity [31]. Furthermore, physical activity has been identified as a key component in the prevention and management of SO through its positive effects on skeletal muscle preservation, reduced fat accumulation, and metabolic regulation [32]. This might partly explain the reason why there were significant interactions of physical activity levels with certain body composition phenotypes concerning albumin, lipids, iron status, HOMA-IR, and hs-CRP.

Although some associations seemed inconsistent across physical activity levels, sufficient physical activity was generally associated with lower odds of unfavorable nutritional and metabolic biomarkers. These findings are broadly consistent with the potential beneficial role of physical activity in metabolic health [32].

LALM/W, obesity, and LALM/W-O were consistently associated with adverse metabolic and inflammatory biomarker profiles in all physical activity levels. The presence of insulin resistance and systemic inflammation was found to be the most common abnormal finding among all sarcopenia and obesity types irrespective of the degree of activity. The constant metabolic burden related to muscle mass reduction and fat accumulation was clearly demonstrated [33]. These findings indicate that appropriate physical activity and dietary strategies may play a supportive role in improving insulin sensitivity and reducing systemic inflammation [34].

Muscle loss and impaired muscle quality have also been related to insulin resistance, presumably via an impairment of anabolic signaling processes and skeletal muscle protein synthesis. Moreover, insulin resistance is characterized by autophagy dysfunction and inflammatory status, thereby speeding up the process of muscle breakdown and deterioration [35]. The impact of skeletal muscle protein turnover in regulating metabolism has been identified, with disruptions in either anabolic and catabolic processes contributing to muscle atrophy and metabolic dysfunctions [36]. Skeletal muscle is the primary site of insulin-mediated glucose uptake and plays a principal role in whole-body glucose homeostasis; decreasing in muscle mass may impair glucose disposal and be linked to deteriorating metabolic regulation [37,38]. Declines in muscle mass and function may be linked to reduce physical activity and lower energy expenditure, further impairing insulin resistance and promoting adiposity accumulation [39]. Concurrently, these mechanisms may establish a vicious cycle in which insulin resistance promotes muscle loss, while muscle wasting further aggravates metabolic dysfunction.

Chronic low-grade inflammation is another key biological mechanism linking both sarcopenia and obesity. Increased adiposity, particularly visceral fat accumulation, promotes the release of pro-inflammatory cytokines and adipokines that are linked to systemic inflammation and metabolic dysfunction [40]. The inflammatory conditions associated with muscle catabolism and reduction in muscle protein synthesis, mediated by the pro-inflammatory cytokines, is likely to be one of the factors contributing to the onset and development of sarcopenia [35,41]. Chronic inflammation and altered autophagy linked to insulin resistance may further impair muscle maintenance and muscle quality [41]. These mechanisms suggest a bidirectional link in which obesity-related inflammation contributes to muscle loss, while muscle wasting may further aggravate metabolic dysfunction and systemic inflammation.

In addition, LALM/W-O phenotypes generally revealed stronger associations with adverse nutritional, metabolic, and inflammatory biomarkers than either sarcopenia or obesity alone. This finding suggests that the coexistence of low muscle mass and excess adiposity may exert synergistic negative effects on metabolic health. In addition, the markedly higher odds of elevated HOMA-IR and hs-CRP observed in the LALM/W-O phenotypes support the hypothesis that combined muscle loss and excess adiposity represent a particularly high-risk metabolic dysregulation. Excess adipose tissue may promote inflammatory cytokine production, whereas reduced muscle mass decreases the primary site of insulin-mediated glucose uptake, together creating a vicious cycle that impairs insulin resistance and systemic inflammation [42].

Limitations of this study should be considered. Because of the cross-sectional study design, causal relationships cannot be established. Although significant interactions between physical activity level and selected body composition phenotypes were observed for several nutritional biomarkers, these interactions were not consistently detected across all phenotypes–biomarker combinations. Therefore, the influence of physical activity on the associations between body composition phenotypes and nutritional biomarkers may be biomarker-specific and should be interpreted with caution. Energy intake and carbohydrate intake may directly influence metabolic biomarkers such as triglycerides and HOMA-IR. Adjustment to these dietary factors may have resulted in partial over-adjustment. Although these variables were included to account for potential confounding related to overall dietary intake and dietary composition, residual concerns regarding over-adjustment cannot be entirely excluded. Physical activity was evaluated using self-reported information, which is exposed to recall bias and potential misclassification. Moreover, individuals classified as completely inactive (0 MET-min/week) may not truly represent entirely inactive individuals. The observation that odds of associations were mostly lower in the inactive group compared with the insufficiently or sufficiently active groups may be explained by limitations in physical activity measurement. Furthermore, the relatively small sample size of the insufficiently active group may have contributed to unstable estimates, as reflected by the wide confidence intervals observed for some associations. However, comparisons between insufficiently active and sufficiently active individuals consistently demonstrated lower odds of adverse nutritional biomarkers among sufficiently active individuals, suggesting that sufficient physical activity may improve metabolic and nutritional conditions. Therefore, the findings should be interpreted cautiously. Standardized assessment of physical activity should be integrated in future studies to improve the accuracy of activity assessment and reduce potential misclassification. The present study used established anthropometric and body composition criteria to classify obesity and low muscle mass. Low muscle mass was defined using ALM/W cutoffs [13], which identifies weight-adjusted muscle mass indices as an alternative to the commonly used height-adjusted ASMI (ALM/height^2^) for defining low muscle mass in the context of varying adiposity. These observed associations must therefore be interpreted cautiously within the context of overlapping body composition models. Although various alternative anthropometric indices have shown some associations with body composition and health outcomes [43,44], it remains uncertain whether they improve upon standard methods for nutritional biomarkers, and further studies are warranted to clarify this. Given the large number of biomarkers and subgroup analysis performed, some findings may have occurred by chance. These results should therefore be interpreted cautiously and considered as exploratory evidence that may help inform future studies rather than definitive conclusions.

## 5. Conclusions

In this cross-sectional study of U.S. adults, low muscle mass, obesity, and their coexistence were all associated with an adverse biomarker, specifically in dyslipidemia, insulin resistance (HOMA-IR), and systemic inflammation (hs-CRP). Most associations were observed among individuals with the combined presence of low muscle mass and obesity, suggesting a more adverse metabolic profile when reduced lean mass coexists with obesity. These findings highlight the important link between body composition, nutritional state, and metabolic status. However, given the cross-sectional design, causal inferences cannot be established. The observed associations should be interpreted as hypothesis-generating rather than causal. Future longitudinal studies are required to clarify associations and to determine whether improvements in body composition and physical activity levels can beneficially influence nutritional and metabolic biomarkers.

## Figures and Tables

**Table 1:** Weighted characteristics among U.S. adults in the NHANES study.

Characteristic (%)		Total (N = 1723)		Inactive (n = 512)		Insufficient (n = 163)		Sufficient (n = 1048)	
		Est	SE	Est	SE	Est	SE	Est	SE
Age	20–39	49.7	1.9	44.5	2.8	44.0	5.0	52.3	2.6
	40–59	50.3	1.9	55.5	2.8	56.0	5.0	47.7	2.6
Sex	Men	44.8	1.5	35.6	2.7	46.3	5.4	48.0	2.0
	Women	55.2	1.5	64.4	2.7	53.7	5.4	52.0	2.0
Race	Mexican American	10.7	2.0	14.8	2.8	9.6	2.5	9.3	1.9
	Other Hispanic	8.6	1.1	12.3	2.4	6.8	1.8	7.4	1.1
	Non-Hispanic White	58.1	3.0	47.8	4.6	58.3	5.7	61.9	3.2
	Non-Hispanic Black	10.2	1.4	12.3	2.3	10.6	2.1	9.4	1.4
	Non-Hispanic Asian	7.8	1.0	9.5	1.6	13	2.7	6.4	1.0
	Other Race	4.6	0.9	3.3	0.9	1.7	1.0	5.5	1.1
^1^ Income	<$20,000	10.8	0.8	15.1	1.5	11.3	3.8	9.2	0.9
	≥$20,000	84.4	1.1	79.7	2.1	84.7	3.4	86.2	1.2
Alcohol	Yes	11.7	1.2	15.1	2.1	7.7	2.1	11.0	1.6
	No	74.3	1.7	66.4	2.6	77.3	4.1	76.8	2.1
Smoke	Yes	25.0	1.8	29.3	3.1	18.6	3.7	24.3	2.2
	No	70.8	1.8	66.4	3.3	78.1	3.7	71.4	2.2
Education	Low (<High school)	11.2	1.4	20.4	2.4	7.5	2.0	8.4	1.3
	Medium	52.2	2.4	53.9	3.1	49.4	4.7	51.9	3.1
	High (≥College)	36.6	3.0	25.6	3.5	43.1	4.7	39.7	3.6
Marriage	Married/Partnered	62.9	2.0	62.9	3.2	63.4	5.0	62.8	2.5
	Previously married	12.6	1.4	16.2	2.3	9.8	2.3	11.6	1.6
	Never married	24.6	1.7	20.9	2.6	26.8	4.9	25.6	2.2
Morbidity	0 disease	52	1.9	47.6	3.1	62.2	6.1	52.3	2.1
	<2 diseases	27.6	1.3	27.6	2.2	20.5	4.3	28.5	1.7
	≥2 diseases	20.4	1.6	24.8	2.1	17.3	4.6	19.2	2.0
Energy	Mean (kcal) *	2055.9	26.3	1943.9	42.6	2011.6	67.8	2103.6	33.2
^2^ Carb	Mean (g) *	240.0	3.3	233.4	5.8	234.0	9.6	243.3	4.9

^1^ Income = Annual household income. ^2^ Carb = Carbohydrate. * This variable is based on an unweighted sample of n = 1416.

**Table 2:** Weighted distribution of body composition phenotypes and nutritional biomarkers according to physical activity level among U.S. adults in NHANES (N = 1723).

Characteristic		Total (N = 1723)		Inactive (n = 512)		Insufficient (n = 163)		Sufficient (n = 1048)	
		Est	SE	Est	SE	Est	SE	Est	SE
Body composition									
ALM/W (<28.27% for men, <23.47% for women)	Low	28.7	2.2	37.5	3.4	35.5	5.3	24.5	2.5
	High	71.3	2.2	62.5	3.4	64.5	5.3	75.5	2.5
BMI (obesity ≥ 30 kg/m^2^)	<18.5	2.4	0.5	2.8	1.0	2.6	1.7	2.2	0.6
	18.5–24.9	31.1	1.8	27.1	2.4	27.9	5.0	33.0	2.5
	25–29.9	29.4	1.4	31.0	3.4	29.8	4.7	28.8	1.7
	≥30	37.1	2.1	39.1	3.1	39.6	5.1	36.0	2.6
WC (≥102 cm for men, ≥88 cm for women)	Low	48.7	2.4	42.9	3.4	48.4	4.7	50.9	2.8
	High	51.3	2.4	57.1	3.4	51.6	4.7	49.1	2.8
FM% (>25% for men, >32% for women)	Low	25.7	2.1	15.3	2.1	17.3	3.0	30.7	2.9
	High	74.3	2.1	84.7	2.1	82.7	3.0	69.3	2.9
LALM/W-O1 (L ALM/W and H BMI)	LALM/W-O1	19.6	1.7	22.9	2.9	21.2	5.2	18.2	2.0
	Normal	27.6	1.7	21.0	2.5	20.2	3.5	31.1	2.5
LALM/W-O2 (L ALM/W and H WC)	LALM/W-O2	24.5	2.0	31.3	3.6	26.5	4.7	21.7	2.2
	Normal	44.6	2.5	36.7	3.4	39.4	4.7	48.2	3.1
LALM/W-O3 (L ALM/W and H FM%)	LALM/W-O3	28.7	2.2	37.5	3.4	35.5	5.3	24.5	2.5
	Normal	25.7	2.1	15.3	2.1	17.3	3.0	30.7	2.9
Nutritional biomarkers									
Albumin (<3.5 g/dL)	Low	1.5	0.4	1.4	0.5	0.8	0.5	1.7	0.6
	High	98.5	0.4	98.6	0.5	99.2	0.5	98.3	0.6
Vitamin D (<20 ng/m)	Low	25.3	1.9	31.3	2.9	39.1	4.8	21.3	2.0
	High	74.7	1.9	68.7	2.9	60.9	4.8	78.7	2.0
Triglyceride (≥150 mg/dL)	Low	73.7	1.5	70.8	2.3	64.2	5.3	76.0	2.2
	High	26.3	1.5	29.2	2.3	35.8	5.3	24.0	2.2
Cholesterol (≥200 mg/dL)	Low	61.2	1.4	56.6	2.9	61.7	4.7	62.8	2.1
	High	38.8	1.4	43.4	2.9	38.3	4.7	37.2	2.1
LDL cholesterol (≥100 mg/dL)	Low	37.1	1.6	31.1	2.3	38.6	4.3	39.1	2.0
	High	62.9	1.6	68.9	2.3	61.4	4.3	60.9	2.0
Iron (<60 µg/dL)	Low	20.3	1.1	25.1	2.6	21.1	2.7	18.4	1.7
	High	79.7	1.1	74.9	2.6	78.9	2.7	81.6	1.7
HOMA-IR (≥2.5)	Low	57.0	1.9	51.3	2.6	51.4	5.3	59.9	2.5
	High	43.0	1.9	48.7	2.6	48.6	5.3	40.1	2.5
hs-CRP (>3 mg/L)	Low	69.2	1.8	62.4	2.6	76.8	4.5	70.6	2.3
	High	30.8	1.8	37.6	2.6	23.2	4.5	29.4	2.3

Abbreviations: ALM/W: Appendicular lean mass per weight, BMI: Body mass index, FM%: Body fat percentage, H: High, HOMA-IR: Homeostasis Model Assessment of Insulin Resistance, hs-CRP: High-sensitivity C-Reactive Protein, LDL: Low-Density Lipoprotein, L: Low, LALM/W-O: Low appendicular lean mass per weight with obesity, WC: Waist circumference.

**Table 3:** Crude and adjusted odds ratios (ORs) with 95% confidence intervals for LALM/W associated with nutritional biomarkers.

Nutritional Biomarkers (1 = Model 1, 2 = Model 2)		Total (n = 1723)		Inactive (n = 512)		Insufficiently Active (n = 163)		Sufficiently Active (n = 1048)	
		OR	95% CI	OR	95% CI	OR	95% CI	OR	95% CI
L Albumin	1	5.3 *	1.6–17.8	4.4 *	1.3–15.4	5.6	0.4–70.8	6.1 *	1.4–27.0
	2	3.0 *	1.1–8.7	6.2	0.8–49.1	NE	NE	4.6 *	1.9–11.2
L Vitamin D	1	1.2	0.9–1.7	0.7	0.5–1.0	0.9	0.3–2.6	1.6 *	1.0–2.5
	2	1.6 *	1.1–2.4	0.9	0.5–1.6	1.6	0.5–5.1	2.1 *	1.3–3.3
H Triglyceride	1	1.6 *	1.2–2.2	1.5	0.9–2.6	0.6	0.3–1.4	1.9 *	1.4–2.7
	2	1.8 *	1.3–2.6	1.1	0.6–2.0	1.7	0.6–4.9	2.4 *	1.5–3.9
H Cholesterol	1	1.4 *	1.1–1.9	1.7 *	1.2–2.5	1.9	0.9–4.2	1.2	0.8–1.9
	2	1.1	0.8–1.6	1.2	0.8–2.0	1.2	0.4–3.4	1.0	0.6–1.5
H LDL	1	1.5 *	1.1–2.0	1.2	0.8–1.7	1.5	0.5–4.5	1.5	1.0–2.4
	2	1.3	0.9–1.9	1.0	0.6–1.8	1.2	0.3–4.4	1.4	0.9–2.1
L Iron	1	1.9 *	1.4–2.6	1.4	0.9–2.4	2.4	0.9–7.0	2.0 *	1.3–3.2
	2	1.3	0.9–1.9	1.3	0.7–2.3	1.2	0.3–4.6	1.3	0.8–2.2
H HOMA-IR	1	3.3 *	2.3–4.6	1.9 *	1.2–3.1	3.2 *	1.2–9.1	4.1 *	2.7–6.3
	2	3.5 *	2.3–5.2	1.5	0.9–2.6	10.1 *	3.8–27.2	4.6 *	2.7–7.7
H hs-CRP	1	4.4 *	3.4–5.8	2.7 *	1.4–5.1	4.5	1.7–12.0	5.7 *	4.0–8.0
	2	3.2 *	2.3–4.5	2.2 *	1.1–4.4	3.7	0.8–17.2	4.1 *	2.7–6.3

Abbreviations: H: High, HOMA-IR: Homeostasis Model Assessment of Insulin Resistance, hs-CRP: High-sensitivity C-Reactive Protein, L: Low, LDL: Low-Density Lipoprotein, NE: Not estimable (Certain estimates were not estimable because of small sample sizes in specific subgroups, limiting statistical analysis). Note: Model 1 = Unadjusted model, Model 2 = Fully adjusted for all covariates (Age, Sex, Race, Education, Marital status, Annual income, Alcohol, Smoke, Multimorbidity, Energy intake, Carbohydrate). * Significant if 95% CI does not include 1.0.

**Table 4:** Crude and adjusted odds ratios (ORs) with 95% confidence intervals for obesity (high BMI, WC, and FM%) associated with nutritional biomarkers.

Nutritional Biomarkers (1 = Model 1, 2 = Model 2)		Total (n = 1723)		Inactive (n = 512)		Insufficiently Active (n = 163)		Sufficiently Active (n = 1048)	
		OR	95% CI	OR	95% CI	OR	95% CI	OR	95% CI
High BMI									
L Albumin	1	7.9 *	2.3–26.4	4.3	0.8–22.9	NE	NE	9.8 *	1.9–50.6
	2	4.4 *	1.2–15.7	2.2	0.4–12.0	NE	NE	7.3 *	1.3–39.6
L Vitamin D	1	2.1 *	1.5–3.0	0.9	0.5–1.8	2.8	1.0–8.3	2.9 *	1.9–4.4
	2	2.3 *	1.6–3.2	1.1	0.5–2.4	4.6 *	1.1–19.9	3.1 *	2.04.8
H Triglyceride	1	5.6 *	3.7–8.6	4.8 *	2.2–10.3	3.9 *	1.2–13.4	6.4 *	3.7–11.0
	2	5.3 *	3.3–8.4	4.7 *	2.1–10.4	3.0	0.6–14.6	5.9 *	3.3–10.7
H Cholesterol	1	1.3	0.9–2.1	1.5	0.8–3.0	2.3	0.8–6.5	1.2	0.7–2.1
	2	1.3	0.8–2.1	1.1	2.5–2.5	2.3	0.7–8.1	1.1	0.6–2.2
H LDL	1	2.0 *	1.4–2.9	1.9 *	1.0–3.5	3.3 *	1.3–8.0	1.9 *	1.2–3.0
	2	1.9 *	1.3–2.9	1.8	0.8–3.7	2.3	0.8–7.3	1.7 *	1.0–3.0
L Iron	1	1.9 *	1.3–2.8	1.6	0.8–3.1	2.9	0.8–10.1	1.9 *	1.2–3.2
	2	2.0 *	1.3–3.2	1.7	0.8–3.6	9.0 *	1.4–59.2	2.0 *	1.1–3.8
H HOMA-IR	1	16.8 *	10.5–26.8	15.9 *	8.8–28.9	32.0 *	5.7–178.0	15.9 *	8.9–28.2
	2	16.9 *	9.6–29.6	18.2 *	8.7–38.1	43.2 *	7.8–237.4	15.1 *	8.1–28.3
H hs-CRP	1	8.3 *	5.6–12.5	8.1 *	4.2–15.4	1.6	0.3–7.3	10.9 *	6.1–19.4
	2	6.5 *	4.1–10.2	6.8 *	3.2–14.4	1.7	0.4–8.3	8.4 *	4.7–14.8
High WC									
L Albumin	1	13.5 *	3.9–47.2	4.6	0.9–25.0	NE	NE	21.0 *	3.8–116.1
	2	5.6 *	1.4–21.8	1.4	0.3–7.2	4.9	0.3–69.6	11.8 *	1.9–73.0
L Vitamin D	1	1.4	0.9–1.9	0.9	0.5–1.4	1.4	0.6–3.6	1.6 *	1.1–2.4
	2	1.7 *	1.2–2.4	1.2	0.7–2.2	2.7	0.8–9.3	1.9 *	1.3–2.9
H Triglyceride	1	2.3 *	1.8–3.1	1.8 *	1.1–3.1	2.2	0.9–5.4	2.6 *	1.7–3.8
	2	2.8 *	2.0–3.9	2.0 *	1.2–3.6	3.3	0.8–13.7	3.0 *	2.0–4.7
H Cholesterol	1	1.6 *	1.2–2.2	1.6 *	1.1–2.5	2.5 *	1.0–6.1	1.5 *	1.0–2.3
	2	1.4	0.9–2.0	1.3	0.7–2.6	1.9	0.7–5.2	1.3	0.8–2.2
H LDL	1	1.8 *	1.4–2.3	1.4	0.9–2.1	2.1 *	1.1–4.0	1.9 *	1.3–2.6
	2	1.7 *	1.3–2.2	1.6	0.8–3.1	1.6	0.6–4.0	1.7 *	1.2–2.4
L Iron	1	1.9 *	1.4–2.7	1.9 *	1.0–3.6	4.0 *	1.4–11.7	1.7 *	1.1–2.8
	2	1.5	0.9–2.4	1.4	0.6–3.1	5.1 *	1.1–23.1	1.4	0.8–2.7
H HOMA-IR	1	7.1 *	5.1–9.9	5.1 *	3.6–7.4	7.3 *	3.2–16.9	8.0 *	5.2–12.1
	2	9.1 *	6.5–12.8	5.7 *	3.6–9.0	12.8 *	3.7–44.4	11.1 *	7.1–17.3
H hs-CRP	1	5.4 *	4.1–7.1	5.4 *	3.0–9.5	2.2	0.7–6.9	6.0 *	4.1–8.8
	2	4.2 *	3.0–5.7	4.4 *	2.5–7.8	1.8	0.5–6.7	4.8 *	3.2–7.1
High FM%									
L Albumin	1	NE	NE	NE	NE	NE	NE	NE	NE
	2	NE	NE	NE	NE	NE	NE	NE	NE
L Vitamin D	1	1.4	0.8–2.5	0.7	0.4–1.3	1.4	0.4–5.5	1.6	0.9–2.8
	2	1.7	0.9–3.3	1.2	0.5–3.0	2.1	0.6–7.9	1.7	0.9–3.3
H Triglyceride	1	3.5 *	2.5–4.9	2.2 *	1.2–3.9	3.4	0.9–12.3	3.7 *	2.4–5.8
	2	4.2 *	2.7–6.6	3.0 *	1.4–6.4	13.7 *	2.7–69.6	3.7 *	2.2–6.4
H Cholesterol	1	1.6 *	1.1–2.3	1.6	0.7–3.6	2.9 *	1.0–8.5	1.5	0.9–2.5
	2	1.4	0.8–2.3	1.1	0.4–3.6	4.2 *	1.2–15.3	1.3	0.6–2.6
H LDL	1	2.2 *	1.5–3.2	1.4	0.7–2.7	4.9 *	2.2–10.9	2.2 *	1.4–3.5
	2	2.3 *	1.6–3.3	1.2	0.6–2.4	9.6 *	3.5–26.4	2.3 *	1.4–3.6
L Iron	1	1.4	0.9–2.1	2.1 *	1.2–3.8	2.0	0.5–7.7	1.2	0.7–2.0
	2	1.1	0.6–1.8	1.7	0.7–4.0	2.5	0.4–13.5	0.9	0.4–1.8
H HOMA-IR	1	8.2 *	5.5–12.3	8.3 *	4.5–15.3	7.4 *	2.0–28.1	8.1 *	4.8–13.8
	2	10.4 *	6.2–17.6	12.4 *	5.4–28.1	16.5 *	3.3–83.1	10.0 *	5.2–18.9
H hs-CRP	1	7.1 *	4.4–11.5	6.3 *	3.4–11.8	29.9 *	3.5–256.6	7.0 *	3.6–13.6
	2	5.7 *	3.1–10.5	7.1 *	3.1–16.2	13.5 *	1.3–142.5	5.5 *	2.5–12.3

Abbreviations: H: High, HOMA-IR: Homeostasis Model Assessment of Insulin Resistance, hs-CRP: High-sensitivity C-Reactive Protein, L: Low, LDL: Low-Density Lipoprotein, NE: Not estimable (Certain estimates were not estimable because of small sample sizes in specific subgroups, limiting statistical analysis). Note: Model 1 = Unadjusted model, Model 2 = Fully adjusted for all covariates (Age, Sex, Race, Education, Marital status, Annual income, Alcohol, Smoke, Multimorbidity, Energy intake, Carbohydrate). * Significant if 95% CI does not include 1.0.

**Table 5:** Crude and adjusted odds ratios (ORs) with 95% confidence intervals for LALM/W-O1-3 (low ALM/W with high BMI or WC, or FM%) associated with nutritional biomarkers.

Nutritional Biomarkers		Total (n = 1723)		Inactive (n = 512)		Insufficiently Active (n = 163)		Sufficiently Active (n = 1048)	
				OR	95% CI	OR	95% CI	OR	95% CI
LALM/W-O1 (Low ALM/W + High BMI)									
L Albumin	1	42.1 *	4.4–405.1	NE	NE	NE	NE	35.7 *	3.7–343.2
	2	19.5 *	1.9–202.4	NE	NE	NE	NE	25.2 *	2.8–230.0
L Vitamin D	1	2.1 *	1.3–3.4	0.7	0.3–1.4	2.7	0.8–9.0	3.0 *	1.8–5.1
	2	2.8 *	1.7–4.5	0.9	0.4–2.1	5.0 *	1.1–22.4	4.1 *	2.3–7.2
H Triglyceride	1	7.5 *	4.7–12.2	6.5 *	2.9–14.7	3.5	0.6–20.1	8.3 *	4.2–16.4
	2	8.2 *	4.5–14.7	4.6 *	1.8–11.5	7.2 *	1.3–40.1	10.8 *	4.5–26.0
H Cholesterol	1	1.6	0.9–2.5	1.8	0.8–4.0	3.6 *	1.1–11.5	1.3	0.7–2.5
	2	1.3	0.7–2.4	1.3	0.5–3.4	2.2	0.4–11.0	1.1	0.5–2.2
H LDL	1	2.4 *	1.4–3.8	1.7	0.8–3.4	4.2	0.8–21.7	2.3 *	1.3–4.2
	2	2.2 *	1.3–3.7	1.6	0.6–3.9	2.5	0.4–15.0	2.1 *	1.1–3.9
L Iron	1	2.4 *	1.7–3.5	1.8	0.9–3.3	2.8	0.6–13.2	2.6 *	1.4–4.7
	2	2.0 *	1.3–3.2	1.8	0.9–3.6	3.4	0.5–21.4	2.0	1.0–4.3
H HOMA-IR	1	23.3 *	11.9–45.7	17.9 *	7.3–43.7	49.9 *	7.1–348.6	22.7 *	9.9–52.3
	2	24.9 *	10.7–57.8	16.3 *	6.1–43.3	151.8 *	22.8–1009.7	26.0 *	9.3–72.2
H hs-CRP	1	16.6 *	10.0–27.5	11.2 *	5.3–23.5	6.7 *	1.0–46.3	21.6 *	11.2–41.4
	2	11.4 *	6.4–20.2	9.4 *	3.6–24.7	5.2	0.6–43.9	15.2 *	7.5–30.4
LALM/W-O2 (Low ALM/W + High WC)									
L Albumin	1	34.6 *	6.4–188.6	NE	NE	NE	NE	31.4 *	5.6–175.4
	2	12.2 *	2.2–67.3	NE	NE	NE	NE	18.3 *	2.7–125.0
L Vitamin D	1	1.4	0.9–2.2	0.7	0.4–1.2	1.3	0.4–3.8	1.9 *	1.2–3.1
	2	2.1 *	1.3–3.4	1.2	0.52.5	3.0	0.6–13.9	2.8 *	1.6–4.8
H Triglyceride	1	2.5 *	1.8–3.5	2.0 *	1.0–3.9	1.1	0.3–3.7	3.0 *	1.9–4.7
	2	3.4 *	2.2–5.2	1.8 *	0.8–3.7	6.8	1.0–47.8	4.3 *	2.4–7.7
H Cholesterol	1	1.8 *	1.2–2.6	2.0 *	1.2–3.3	3.2 *	1.2–8.3	1.5	0.9–2.6
	2	1.4	0.8–2.3	1.4	0.7–3.0	1.9	0.5–6.6	1.2	0.6–2.2
H LDL	1	1.9 *	1.3–2.8	1.5	0.9–2.3	2.1	0.7–6.9	2.0 *	1.3–3.3
	2	1.8 *	1.2–2.7	1.4	0.6–2.9	1.6	0.3–7.6	1.9 *	1.2–3.0
L Iron	1	2.5 *	1.7–3.6	2.1 *	1.2–3.7	5.1 *	1.3–19.7	2.4 *	1.4–4.1
	2	1.7 *	1.1–2.7	1.7	0.9–3.3	2.4	0.6–10.6	1.6	0.8–3.2
H HOMA-IR	1	9.4 *	6.2–14.2	5.1 *	3.4–7.6	15.7 *	5.1–48.0	11.3 *	6.4–20.1
	2	14.4 *	8.8–23.7	5.2 *	2.9–9.5	84.4 *	18.9–377.2	20.1 *	9.9–40.7
H hs-CRP	1	9.9 *	7.1–13.8	7.2 *	3.6–14.5	6.0 *	1.5–24.3	12.4 *	7.9–19.4
	2	7.2 *	4.9–10.8	6.0 *	2.8–12.9	4.8	0.8–28.4	9.2 *	5.6–15.0
LALM/W-O3 (Low ALM/W + High FM%)									
L Albumin	1	NE	NE	NE	NE	NE	NE	NE	NE
	2	NE	NE	NE	NE	NE	NE	NE	NE
L Vitamin D	1	1.5	0.8–2.3	0.6	0.3–1.2	1.3	0.3–5.9	1.9 *	1.0–3.7
	2	2.2 *	1.1–4.7	1.1	0.4–3.3	2.8	0.6–14.6	2.7 *	1.3–5.6
H Triglyceride	1	3.8 *	2.6–5.6	2.5 *	1.2–5.1	2.1	0.5–9.0	4.5 *	2.8–7.3
	2	5.3 *	3.1–9.2	2.9 *	1.2–7.0	19.8 *	1.6–239.8	5.8 *	2.9–11.6
H Cholesterol	1	1.8 *	1.2–2.7	2.0	0.9–4.6	3.8 *	1.2–11.4	1.5	0.8–2.9
	2	1.4	0.8–2.5	1.3	0.4–4.3	4.1	0.9–19.0	1.2	0.5–2.7
H LDL	1	2.3 *	1.6–3.5	1.4	0.7–2.8	4.7 *	1.6–13.9	2.4 *	1.4–4.1
	2	2.4 *	1.5–3.8	1.2	0.5–2.9	8.6 *	2.3–32.7	2.4 *	1.4–4.2
L Iron	1	2.1 *	1.5–2.9	2.4 *	1.3–4.5	3.0	0.7–13.8	1.9 *	1.1–3.2
	2	1.3	0.8–2.1	1.9	0.7–4.9	2.6	0.5–12.9	1.1	0.5–2.4
H HOMA-IR	1	12.5 *	8.0–19.4	9.6 *	4.9–18.7	12.0 *	2.5–57.7	14.0 *	8.0–24.5
	2	19.6 *	10.7–36.1	12.9 *	5.1–32.3	150.0 *	16.5–1366.4	23.1 *	11.0–48.8
H hs-CRP	1	13.5 *	8.2–22.3	9.3 *	4.0–21.7	53.9 *	5.7–506.4	15.8 *	7.7–32.3
	2	10.2 *	5.3–19.6	10.0 *	3.8–26.0	34.8 *	2.2–540.6	11.9 *	4.9–28.9

Abbreviations: H: High, HOMA-IR: Homeostasis Model Assessment of Insulin Resistance, hs-CRP: High-sensitivity C-Reactive Protein, L: Low, LDL: Low-Density Lipoprotein, NE: Not estimable (Certain estimates were not estimable because of small sample sizes in specific subgroups, limiting statistical analysis). Note: Model 1 = Unadjusted model, Model 2 = Fully adjusted for all covariates (Age, Sex, Race, Education, Marital status, Annual income, Alcohol, Smoke, Multimorbidity, Energy intake, Carbohydrate). * Significant if 95% CI does not include 1.0.

## Data Availability

The data used in this study is publicly available from the National Health and Nutrition Examination Survey (NHANES) database. https://www.cdc.gov/nchs/nhanes/index.html (accessed on 5 January 2026).

## Supplementary Materials

The following supporting information can be downloaded at: https://www.mdpi.com/article/10.3390/ijerph23060815/s1. Table S1. Interaction between body composition phenotypes and physical activity level on nutritional biomarkers.

## Abbreviations

ALM	Appendicular Lean Mass
ALM/W	Appendicular Lean Mass per Body Weight
BMI	Body Mass Index
CDC	Centers for Disease Control and Prevention
CI	Confidence Interval
COPD	Chronic Obstructive Pulmonary Disease
DXA	Dual-Energy X-ray Absorptiometry
Est	Estimate
EWGSOP	European Working Group on Sarcopenia in Older People
FM%	Body Fat Percentage
HOMA-IR	Homeostatic Model Assessment for Insulin Resistance
hs-CRP	High-Sensitivity C-Reactive Protein
LALM/W-O	Low Appendicular Lean Mass per Body Weight (ALM/W) with Obesity
LALM/W-O1	Low ALM/W with High BMI
LALM/W-O2	Low ALM/W with High Waist Circumference
LALM/W-O3	Low ALM/W with High Body Fat Percentage
LDL	Low-Density Lipoprotein
MET	Metabolic Equivalent
NHANES	National Health and Nutrition Examination Survey
OR	Odds Ratio
SE	Standard Error
U.S.	United States
WC	Waist Circumference
