# Supplementary material for: Association Between Nutritional Biomarkers and Low Muscle Mass, Obesity, and Low Muscle Mass with Obesity Across Physical Activity Levels Among U.S. Adults: Finding from the National Health and Nutrition Examination Survey 2015–2018
Source: Int J Environ Res Public Health. 2026 Jun 19;23(6):815. doi: 10.3390/ijerph23060815 (PMC13300325; doi:10.3390/ijerph23060815)
Supplement: Supplementary file 1 [file ijerph-23-00815-s001.zip › ijerph-4339094-supplementary.pdf]

Table S1. Interaction between body composition phenotypes and physical activity level on nutritional biomarkers.

Nutritional biomarker	LALM/W	BMI-defined obesity	WC-defined obesity	FM%-defined obesity	LALM/W-O1	LALM/W-O2	LALM/W-O3
Albumin	P=0.70	P<0.001	P<0.001	P=0.36	P<0.001	P<0.001	P=0.73
Vitamin D	P=0.16	P=0.17	P=0.49	P=0.71	P=0.48	P=0.20	P=0.54
Triglycerides	P=0.16	P<0.001	P=0.70	P=0.40	P<0.001	P=0.44	P=0.17
Total cholesterol	P=0.61	P<0.001	P=0.51	P=0.14	P<0.001	P=0.93	P=0.32
LDL cholesterol	P=0.45	P<0.001	P=0.50	P<0.001	P<0.001	P=0.70	P=0.02
Iron	P=0.71	P<0.001	P=0.27	P=0.28	P<0.001	P<0.001	P=0.62
HOMA-IR	P=0.05	P=0.86	P=0.42	P=0.59	P=0.25	P=0.02	P=0.36
hs-CRP	P=0.74	P<0.001	P=0.38	P=0.43	P<0.001	P=0.91	P=0.71

Abbreviations: BMI: Body mass index, FM%: Body fat percentage, H: High, HOMA-IR: Homeostasis Model Assessment of Insulin Resistance, hs-CRP: High-sensitivity C-Reactive Protein, LALM/W: Low appendicular lean mass per body weight, LALM/W-O: Low appendicular lean mass per body weight with obesity, LDL: Low-Density Lipoprotein, L: Low, WC: Waist circumference

P-values represent tests of interaction between body composition phenotype and physical activity level obtained from survey-weighted logistic regression models accounting for the complex NHANES sampling design.
